# Biophysical Mechanisms of Vaginal Smooth Muscle Contraction: The Role of the Membrane Potential and Ion Channels

**DOI:** 10.3390/pathophysiology31020018

**Published:** 2024-05-14

**Authors:** Chitaranjan Mahapatra, Ravinder Kumar

**Affiliations:** 1 Cardiovascular Research Institute, University of California San Francisco, San Francisco, CA 94158, USA; 2 Paris Saclay Institute of Neuroscience, 91440 Saclay, France; 3 Department of Pathology, College of Medicine, University of Tennessee Health Science Center, Memphis, TN 38163, USA

**Keywords:** vagina contraction, smooth muscle, biophysics, ion channel, calcium dynamics, membrane potential

## Abstract

The vagina is an essential component of the female reproductive system and is responsible for providing female sexual satisfaction. Vaginal smooth muscle contraction plays a crucial role in various physiological processes, including sexual arousal, childbirth, and urinary continence. In pathophysiological conditions, such as pelvic floor disorders, aberrations in vaginal smooth muscle function can lead to urinary incontinence and pelvic organ prolapse. A set of cellular and sub-cellular physiological mechanisms regulates the contractile properties of the vaginal smooth muscle cells. Calcium influx is a crucial determinant of smooth muscle contraction, facilitated through voltage-dependent calcium channels and calcium release from intracellular stores. Comprehensive reviews on smooth muscle biophysics are relatively scarce within the scientific literature, likely due to the complexity and specialized nature of the topic. The objective of this review is to provide a comprehensive description of alterations in the cellular physiology of vaginal smooth muscle contraction. The benefit associated with this particular approach is that conducting a comprehensive examination of the cellular mechanisms underlying contractile activation will enable the creation of more targeted therapeutic agents to control vaginal contraction disorders.

## 1. Introduction

The vagina is a female reproductive organ that undergoes physiological changes throughout a woman’s existence. It exhibits diverse physiological responses to hormonal fluctuations, such as those observed during puberty, menstruation, pregnancy, and menopause. The vagina is also an essential component of the female reproductive system and is responsible for providing female sexual satisfaction [1]. Smooth muscle is found throughout the body and serves a variety of functions [2]. Smooth muscle is an involuntary, non-striated muscle divided into two subgroups: single-unit and multi-unit smooth muscle. Within single-unit muscle, the whole bundle or sheet of smooth muscle cells contracts as a syncytium [3]. Smooth muscle is found in the walls of hollow organs, including the stomach, intestines, bladder, and uterus, as well as walls of blood vessels and lymph vessels. Smooth muscle contraction is categorized into tonic and phasic types based on their contractile properties. Tonic smooth muscles exhibit slow, sustained contractions that can maintain tension for extended periods, commonly found in structures like the walls of blood vessels. In contrast, phasic smooth muscles display rhythmic contractile activity, contracting more rapidly and intermittently. These are typically found in systems like the gastrointestinal and urogenital tracts, facilitating functions like peristalsis or the rhythmic movement of substances through organs [4].

**Figure 1:**
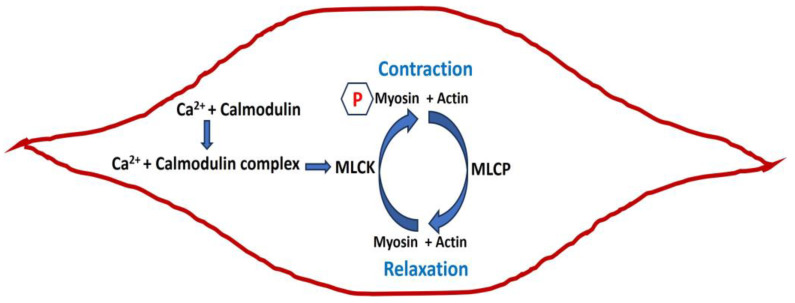
Schematic diagram of smooth muscle in relaxed and contracted states with internal mechanisms.

In smooth muscle contraction, excitation–contraction coupling (E-C coupling) is a fundamental process that links the electrical excitation of the muscle cell membrane (sarcolemma) to muscle contraction [5]. The calcium ions (Ca^2+^) play a central role in activating various proteins involved in the contractile process. The sequence of events in E-C coupling starts with the generation of an action potential on the sarcolemma. This action potential leads to an increase in free Ca^2+^ in the cytoplasm. When Ca^2+^ binds to calmodulin, it undergoes a conformational change, activating Myosin Light Chain Kinase (MLCK). Activated MLCK then phosphorylates the myosin regulatory light chain (RLC), a crucial step that binds the myosin heads to actin filaments. As myosin heads bind to actin, they undergo a conformational change, causing the sliding of actin and myosin filaments past each other. This sliding filament mechanism generates force, resulting in smooth muscle contraction. Concurrently, Myosin Light Chain Phosphatase (MLCP) acts to dephosphorylate the RLC, regulating the balance between contraction and relaxation. Figure 1 illustrates the schematic representation of the internal mechanisms of an isolated smooth muscle cell in both its relaxed and contracted states. The sign P denotes the phosphorylation.

The phasic contraction of vaginal smooth muscle (VSM) is vital for several physiological processes, such as sexual arousal, childbirth, and urinary continence. Additionally, smooth muscle cells within the vaginal wall are essential for preserving tissue integrity and responding to both mechanical and biochemical stimuli [6,7]. A set of cellular and sub-cellular physiological mechanisms regulates the contractile properties of the VSM cells. Ca^2+^ influx is a key determinant of VSM contraction, facilitated through voltage-dependent Ca^2+^ channels (VDCC) and Ca^2+^ release from intracellular stores [2]. The value of intracellular Ca^2+^ concentration is modulated by the coordinated action of ion channels, neurotransmitters, and hormonal signals, leading to cyclic changes in vaginal tone and elasticity [6,8,9]. During sexual arousal, pelvic nerve stimulation triggers the relaxation of vaginal smooth muscles, facilitating intercourse [10]. Disorders related to vaginal smooth muscle manifest when there are irregular contractions or modifications in its mechanical characteristics. Such alterations can profoundly impact vaginal health and function. For instance, a disorder of smooth muscle contraction can lead to an increase in vaginal creep behavior, affecting the viscoelastic response of the vagina, crucial for maintaining structural integrity under prolonged pressure [6]. Additionally, deformations in the vagina resulting from these contractions are pivotal in the onset of pelvic floor disorders, necessitating a deeper understanding of these changes to address their root causes [11]. Moreover, modifications in the mechanical properties of the vagina can induce adverse shifts in its contractile behavior, impacting its ability to contract and relax effectively. After vaginal delivery, smooth muscle hypersensitivity can emerge, potentially jeopardizing the functionality of the urethral sphincter and underscoring the need for vigilant postpartum care [12]. A comprehensive understanding of vaginal smooth muscle dynamics is essential, especially given its critical role in vaginal health. In the context of conditions like pelvic floor disorders, dysfunctions in vaginal smooth muscle can precipitate urinary incontinence and pelvic organ prolapse. Dysregulation of VSM tone and contractility may result from factors such as aging, hormonal changes, childbirth trauma, or neurological disorders [13,14]. Insights into the biophysical mechanisms governing smooth muscle contraction not only enhance our fundamental understanding of muscle physiology but also hold promise for developing novel therapeutic strategies targeting smooth muscle disorders and dysfunctions [15]. Therefore, it is crucial to understand the underlying cellular and sub-cellular biophysical processes in VSM physiology for developing targeted therapeutic interventions to manage pelvic floor disorders and addressing pathological conditions related to reproductive health effectively.

Smooth muscle contraction relies on intricate interactions between cytoskeletal elements, ion channels, hormones, and biochemical and calcium signaling pathways [16,17,18]. As mentioned earlier, Ca^2+^ serves as a critical mediator, initiating cross-bridge cycling between actin and myosin, ultimately leading to force development [19,20]. Ion channels also play a pivotal role in regulating membrane potential, intracellular Ca^2+^ concentration, and, ultimately, smooth muscle contractility [21,22,23]. Furthermore, the roles of sodium (Na^+^), chloride (Cl^−^), and potassium (K^+^) channels in controlling the membrane potential are crucial for deciphering the underlying mechanisms of smooth muscle function and its dysregulation [24,25,26,27]. Therefore, understanding the interplay between membrane potential and ion channels in VSM contraction is paramount for elucidating reproductive physiology and addressing associated disorders. Additionally, the involvement of smooth muscle tissue’s morphological and structural aspects is also imperative for comprehending its contractile properties and functionality [16]. The alterations in the structure and function of VSM, such as those induced by pelvic organ prolapse, underscore the importance of understanding mechanical stimulation in elucidating disease mechanisms [28].

Comprehensive reviews on smooth muscle biophysics are relatively scarce within the scientific literature, likely due to the complexity and specialized nature of the topic. While research articles and studies delve into specific aspects of smooth muscle physiology and biophysics [2], a consolidated overview that encompasses all facets of smooth muscle biophysics is less common. This scarcity may stem from the interdisciplinary nature of biophysics, which requires expertise in both biology and physics [29]. However, the importance of understanding smooth muscle biophysics in various physiological and pathological conditions underscores the necessity for more comprehensive reviews to bridge the gap between basic science and clinical applications. To bridge this divide, the current review aims to enhance our understanding of the biophysical facets of VSM contraction. It includes experimental shreds of evidence for biophysical processes in VSM and other smooth muscles and establishes a model for VSM cell contraction. This understanding offers valuable insights into typical physiological mechanisms, guiding the development of therapeutic interventions for addressing reproductive disorders and promoting optimal women’s health. In electrophysiological experiments, the whole-cell clamp combines features of patch and voltage clamping, enabling simultaneous measurement of currents from multiple ion channels while controlling the cell’s membrane potential [30]. In contrast, the current clamp records membrane potential changes in response to injected current, providing data on the cell’s overall electrical activity and ion channel behavior [31]. Alongside these electrophysiological methods, immunohistochemistry visualizes and localizes specific ion channels within cells using antibodies [32]. This approach complements electrophysiological data by revealing the presence, distribution, and cellular localization of ion channels. Fluorescent microscopy plays a pivotal role in studying Ca^2+^ dynamics in smooth muscle cells by allowing researchers to visualize Ca^2+^ signaling events in real time. One significant technique is total internal reflection fluorescence (TIRF) microscopy, which selectively illuminates and visualizes the area just beneath the cell membrane. This method is particularly useful for observing localized Ca^2+^ signaling events at the plasma membrane interface, where many Ca^2+^ channels and signaling molecules are situated. Another approach involves fluorescence confocal imaging, providing detailed insights into Ca^2+^ signaling mechanisms within the smooth muscle cells [33]. This technique enables the exploration of intracellular Ca^2+^ oscillations and responses to various stimuli, revealing the dynamic nature of calcium signaling in these cells. Measuring tension in smooth muscle involves various techniques and methods, both direct and indirect. One commonly used method is the mechanical measurement of muscle tension, where an external force is applied mechanically to the muscle, and the resulting resistance or tension is recorded [34]. Another approach focuses on the length–tension relationship, where the tension in the muscle is measured at different muscle lengths to determine optimal muscle performance. Comprehensive experimental methods for studying electrophysiology, Ca^2+^ dynamics and force generation mechanisms have not yet been applied to the vaginal smooth muscle. Therefore, this review extrapolates potential mechanisms in VSM cells by comparing them with experimentally verified data from other types of smooth muscle. 

## 2. Materials and Methods

We conducted a thorough investigation using the MEDLINE database via PubMed [35], focusing on English articles published at any time. We aimed to explore the associations among all types of smooth muscle ion channels, biophysics, gap junctions, neurotransmitters, neuromodulators, calcium dynamics, intracellular electrical activities (depolarization, hyperpolarization, action potential, and slow waves), and experimental and computational studies. Then, we meticulously screened all these relevant studies. Exclusion criteria comprised non-English articles, conference/symposium abstracts, and studies duplicating information from other sources. Priority was given to the latest and most comprehensive manuscripts in cases of overlap. Our selection criteria encompassed original research articles, including randomized and non-randomized clinical trials, experimental studies, prospective observational studies, retrospective cohort studies, and case–control studies, along with review articles exploring the potential impact of ion channels on smooth muscle excitability and contraction development. Each included article underwent thorough scrutiny, and supplementary references were consulted to ensure comprehensive coverage. Ultimately, we designed a model workflow diagram to elaborate on all the essential steps necessary for vaginal smooth muscle contraction. 

## 3. Membrane Potential in Smooth Muscle Contraction

The electrical terms used in excitable cells are membrane potential, resting membrane potential (RMP), depolarization, hyperpolarization, action potential (AP), slow wave (SW), and bursting firing patterns [36]. Conduction velocity is also another property to measure the propagation of membrane potential in all types of excitable cells [37]. The membrane potential, a fundamental aspect of cellular physiology, plays a crucial role in various cellular processes beyond merely facilitating ion transport [38]. Recent research highlights its significance in governing diverse cellular functions, including cell cycle regulation, cell volume control, proliferation, and muscle activity [39]. While traditionally associated with the generation of AP in excitable cells, such as neurons and muscle cells, emerging evidence suggests that the RMP exerts profound effects on cell behavior across different cell types [39]. Biophysical signaling mediated by membrane potential serves as a key regulator of long-term cellular behavior, contributing significantly to both excitable and non-excitable cell functions [40]. Furthermore, the membrane potential acts as a pivotal determinant of cell-to-cell signaling and coordination, ultimately influencing broader physiological processes within organisms. It serves as a medium through which cells communicate with each other and the central nervous system, facilitating the transmission of messages critical for maintaining homeostasis and orchestrating various bodily functions [41]. 

**Figure 2:**
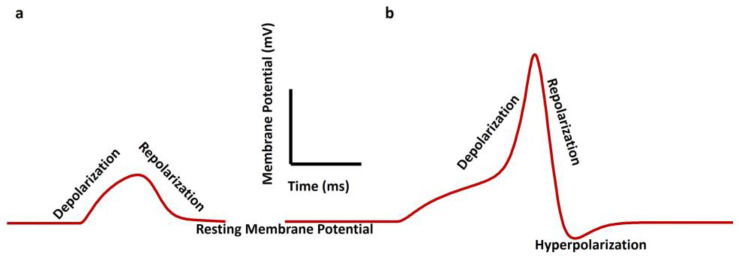
Illustration of slow wave (**a**) and pacemaking type action potential (**b**) in smooth muscle cells.

Smooth muscle cells exhibit distinct patterns of electrical activity, including AP, spontaneous AP, spontaneous depolarizations, spontaneous hyperpolarizations, and SW, which play crucial roles in triggering muscle contraction. APs in smooth muscle cells are characterized by slower kinetics than skeletal muscle, with durations nearly fifty times longer [2]. Spontaneous APs, spontaneous depolarizations, and spontaneous hyperpolarizations are evoked without any external electrical and synaptic input stimulus [15,19]. APs are generated in the urinary bladder [23,42], vas deferens [43,44], urethra [25,45,46], and ureter [47,48,49,50] smooth muscles. Slow waves, rhythmic electrophysiological events in the gastrointestinal (GI) tract, are generated and propagated by interstitial cells of Cajal (ICC), which are electrically coupled to smooth muscle cells [51]. These slow waves organize primary electrical activity in GI smooth muscles and are essential for regulating peristalsis and digestive processes [52]. Figure 2 illustrates SW (a) and pacemaking AP (b) in smooth muscle cells. The RMP is the membrane potential when the cell is not excitable. The depolarization is the positive shift of the membrane potential from the RMP. When the membrane potential returns to the RMP from the peak value of SW/AP, it is known as repolarization. Any negative membrane potential shift from the RMP is known as hyperpolarization.

Table 1 illustrates the value of the RMP and types of electrical properties generated in major important smooth muscle cells. 

Electrical activity in VSM plays a pivotal role in various physiological functions, including sexual arousal and childbirth. Research has shown that electrical stimulation can induce contractions in VSM strips, suggesting the presence of nerve-mediated pathways regulating muscle activity [62]. These contractions are believed to be mediated by cholinergic nerves (muscarinic acetylcholine receptor M3), indicating the involvement of neurotransmitter release in modulating smooth muscle contractility [62]. Furthermore, the electrical properties of VSM contribute to its viscoelastic response, with smooth muscle contraction leading to changes in vaginal creep behavior, highlighting the dynamic nature of electrical activities in regulating tissue mechanics [6]. The membrane potential, primarily controlled by ion channel activity, is a critical determinant of cellular excitability and contractility in VSM cells. Research indicates that changes in membrane potential, induced by various stimuli, such as hormonal fluctuations or mechanical stretch, play a significant role in modulating the responsiveness of vaginal smooth muscle. For instance, depolarization of the smooth muscle cell membrane can increase cellular responsiveness, leading to enhanced contractile activity, while hyperpolarization may reduce smooth muscle relaxation [63]. Unfortunately, like the urinary bladder, ureter, vas deference, and gut smooth muscle, intracellular electrophysiology for VSM cells is not well investigated. Shafik, One lab [64,65] has recorded both extracellular SW and AP bursting from the VSM using the electroretinogram method. They have also suggested that the RMP of the VSM is like that of the uterine smooth muscle, i.e., at −50 mV. The mean velocity for the human VSM cell is mentioned as 4.3 cm/s. Recent studies have explored the therapeutic potential of electrical stimulation for pelvic floor rehabilitation, indicating its predictive role in enhancing muscle contraction force and detrusor function [66]. Moreover, advancements in non-invasive techniques for measuring electrical signals in uterine smooth muscle have contributed to a deeper understanding of the role of electrical activity in reproductive physiology [67]. Pathophysiological conditions can lead to modifications in the signaling cascades involved in initiating and sustaining contraction and relaxation [68].

## 4. Ion Channel Biophysics in Smooth Muscle

Ion channels are integral membrane proteins that regulate the flow of ions across cell membranes, thereby playing fundamental roles in various physiological processes, including neuronal signaling, muscle contraction, and hormone secretion. These channels exhibit diverse structural and functional characteristics, with significant types including voltage-gated, ligand-gated, and mechanically gated ion channels [69]. Voltage-gated ion channels, such as voltage-gated Na^+^ channels, voltage-gated K^+^ channels, voltage-gated Cl^−^ channels, and voltage-gated Ca^2+^ channels, respond to changes in membrane potential, enabling rapid AP generation and propagation in excitable cells, like neurons and muscle cells [21]. Specific neurotransmitters or ligands, such as nicotinic acetylcholine receptors, activate ligand-gated ion channels, leading to ion flux and subsequent cellular responses. Mechanically gated ion channels, like those found in sensory neurons, open in response to physical stimuli, such as pressure or stretching, transducing mechanical signals into electrical signals [70]. Various mechanisms, including protein phosphorylation, protein-protein interactions, and changes in intracellular ion concentrations, tightly regulate the activity of ion channels. Protein phosphorylation, for instance, can modulate ion channel function by altering its conformation or membrane localization [70]. Additionally, intracellular ion concentrations, such as Ca^2+^, play critical roles in regulating ion channel activity. Changes in Ca^2+^ levels can directly influence ion channel gating or indirectly affect channel function by activating Ca^2+^-sensitive signaling pathways [71]. Overall, the intricate regulation of ion channels ensures precise control over cellular excitability and function, highlighting their importance in maintaining physiological homeostasis [72]. Various types of ion channels play crucial roles in regulating membrane potential and intracellular Ca^2+^ concentration in smooth muscle cells. Notably, the differential expression patterns of ion channels and their regulation by signaling pathways can lead to distinct contractile or relaxant responses, influencing muscle tone and contraction [73]. These channels, embedded within the sarcolemma, control the flow of ions across the cell membrane, influencing membrane potential and intracellular Ca^2+^ concentrations, which are critical determinants of muscle contraction [21]. 

Voltage-dependent Na^+^ channels have garnered significant attention in research and are often considered the most extensively studied channel type. Na^+^ channels are integral to the physiological function of smooth muscle, including that of the vagina. In tissues where swift activation is less critical, Na^+^ ions often serve as the secondary carriers of inward current, particularly in many smooth muscles as highlighted by various studies [74]. These channels regulate membrane potential, intracellular calcium concentration, and contractility. Tetrodotoxin (TTX) stands out as a highly specific channel blocker, and sensitivity to TTX helps categorize these channels. However, emerging evidence indicates the presence of authentic Na^+^ channels in certain smooth muscles, like VSM cells. Na^+^ channels in smooth muscles could be either TTX sensitive or TTX insensitive. Still, there are lingering questions about their functionality in healthy tissues versus their potential induction in pathological states [21]. Ca^2+^ plays a pivotal role in activating various cellular pathways, making channels that facilitate Ca^2+^ entry prime targets for drug interventions. Ca^2+^ channels that allow Ca^2+^ ions to flow are vital in tissues like cardiac and smooth muscles, contributing to the inward current during depolarization and action potentials. Among these, L-type and T-type Ca^2+^ channels are important Ca^2+^ channels in all smooth muscles, including the vagina [74]. L-type channels exhibit prolonged open times and high conductance, activated by substantial depolarizations. Conversely, T-type channels are short lived, possess minimal conductance, and respond to minor depolarizations. Common blockers for L-type channels include nifedipine, nicardipine, verapamil, and diltiazem. Subsequent research identified N-type channels, distinct from L and T types, leading to the discovery of additional channels, like P/Q and R types. These channels can be distinguished by their susceptibility to specific toxins, such as ω-conotoxin GVIA for N-type and ω-agatoxin for P/Q-type, and R-type channels show resistance to toxins. Specifically, L-type Ca^2+^ channel antagonists, such as nifedipine, effectively inhibit smooth muscle contraction in the urogenital tract by blocking L-type Ca^2+^ channels. They also prevent the refilling of intracellular Ca^2+^ stores by impeding cholinergic nerve stimulation, where acetylcholine activates muscarinic receptors on the membrane [74]. 

K^+^ channels typically consist of four subunits, which are intricate membrane-spanning proteins. These subunits share a common amino acid sequence in one of their pore-forming loops, determining the channel’s selectivity. Three primary classes of K^+^ channels exist, categorized by the number of transmembrane segments in each subunit: two, four, or six. The 4TM proteins likely contribute to the fundamental K^+^ leak conductance. The 2TM group encompasses an inward rectifier and ATP-sensitive and G-protein-coupled channels, while the 6TM group includes Ca^2+^-activated and delayed rectifier channels. Voltage-gated K^+^ (Kv) channels contribute to repolarization during AP, aiding in maintaining the RMP and controlling excitability [75]. Inward rectifier channels are voltage-gated K^+^ channels found in various excitable cells. The term “rectifiers” describes their ability to allow easier ion flow into the cell than out. This rectification is not due to the channel proteins’ inherent voltage sensitivity but results from large intracellular ions that canot traverse the channel. Inward rectifiers are involved in maintaining the resting membrane potential near the equilibrium potential for potassium (E_K_). Blocking agents include divalent cations, like Mg^2+^, and organic compounds, such as spermine, spermidine, putrescine, and cadaverine. Altering the E_K_ affects the ion driving force, causing a minor outward current. However, when extracellular K^+^ and the driving force change appropriately, an inward current occurs. Voltage-dependent Ca^2+^-activated K^+^ (KCa) channels produce a hyperpolarizing afterpotential, influencing the AP firing rate after they are activated by the intracellular Ca^2+^ and/or membrane potential. Three main types of channels exist based on conductance: small, medium, and large. Large conductance channels (with unit conductance of 400–800 pS), also known as KCa1.1 or Maxi K channels, are crucial for regulating smooth muscle contraction and neuronal excitability. Selective blockers for these channels include charybdotoxin and apamine. KCa1.1 channels play a significant role in mediating the effects of β-adrenoceptor agonists, like adrenaline. These agonists increase cAMP levels, activating protein kinase A and directly stimulating KCa1.1 channels via G-proteins, although the importance of this pathway is debated. Small and intermediate conductance channels are less understood than their large counterparts. Their unit conductances range from 2–20 pS and 20–85 pS, respectively. These channels are more sensitive to intracellular Ca^2+^ levels and are regulated solely by internal Ca^2+^ ions. Intermediate channels are prevalent in smooth muscles, while small channels are more common in the central nervous system. Interestingly, the protein-coding regions of these channels lack consensus calcium-binding motifs [75].

ATP-sensitive K^+^ (K_ATP_) channels are instrumental in managing insulin release from pancreatic β-cells. These channels feature regulatory elements that modulate ion channel activity. When glucose concentrations rise, β-cells absorb and metabolize glucose, elevating intracellular ATP. This surge in ATP suppresses the channel’s regulatory components. As a result, the channels switch off, causing cellular depolarization, an influx of calcium, and subsequent insulin secretion. Moreover, K_ATP_ channels are vital for smooth muscle functionality. They inhibit action potential formation, allowing for subtle membrane potential adjustments. These channels are integral to diverse vascular reactions and are present in various tissues like the brain, heart, pancreas, and vascular smooth muscle. Additionally, K_ATP_ channels influence the baseline membrane permeability of specific smooth muscles. They can also activate in scenarios of metabolic stress, holding significant roles in both normal and abnormal physiological conditions [76]. Delayed rectifier K^+^ (KDR) channels are voltage-sensitive K^+^ channels pivotal for the repolarization of action potentials in smooth muscles. These channels regulate K^+^ outflow, influencing both the duration and frequency of action potentials and, consequently, muscle contraction and relaxation. They were first discovered in cold-blooded creatures, like squids, where they expedite repolarization after voltage-sensitive Na^+^ channels deactivate, ensuring a consistent action potential frequency. Tetraethylammonium (TEA) is a classical pharmacological agent known to block voltage-gated K^+^ channels, including KDR channels. Additionally, scorpion venom contains K^+^ channel blocker toxins, which are short peptides that can also block K^+^ channels. 

Transient receptor potential (TRP) channels are non-selective cation channels present in all eukaryotic cells, playing roles in various functions such as responding to painful stimuli, mediating receptor-induced excitation, and regulating the cell cycle. These channels are often activated or modulated by phosphatidylinositol signal transduction pathways. They are categorized into three main subfamilies: TRPC (canonical), TRPV (vanilloid receptor, osm9-like), and TRPM (melastatin). In the TRPC family, TRPC3, TRPC4, TRPC6, and TRPC7 are predominantly found in smooth and cardiac muscle cells, acting as receptor-activated non-selective cation channels in these tissues [77]. Within the TRPV family, TRPV1 is a calcium-permeable channel activated by heat and reduced pH and inhibited by phosphatidylinositol-4,5-bisphosphate (PIP2). Stretching increases TRPV2 activity, and both TRPV3 and TRPV4 are activated by increased temperature. TRPV4 is sensitive to cell swelling (hypotonicity). TRPV5 and TRPV6 form a subfamily found in the kidneys and intestinal transporting epithelia. The TRPM family includes TRPM1, which is broadly expressed in normal tissues with unspecified functions. TRPM2 is a Ca^2+^-permeable channel activated by ADP ribose and nicotinamide adenine dinucleotide. TRPM3 is constitutively active and calcium permeable, with activity increasing under hypotonic conditions but sharing little similarity with TRPV4. TRPM4 is mainly found in the kidneys and CNS tissues, along with TRPM5, which is the only monovalent-selective channel in the TRP family. These channels are activated through GPCRs linked to PLC-dependent Ca^2+^ release, possibly via direct Ca^2+^ binding. The diverse nature of the TRP channel family makes them promising targets for pharmacological interventions [78]. However, current pharmacology for TRP receptors, aside from TRPV1, is limited, necessitating the development of subtype-specific drugs.

Early research on striated muscle indicated that chloride (Cl^−^) likely distributes passively within this tissue, given its proximity to the membrane potential of around −65 mV. This suggests a significant permeability to chloride, which helps stabilize the resting potential and modulate responses to Cl^−^ channel activation or inhibition. Increased Cl^−^ permeability can lead to tissue depolarization, offering cells an effective mechanism to react to external stimuli. Any genetic abnormalities affecting these channels can lead to severe diseases. Cl^−^ channels play a crucial role in the physiological function of smooth muscle cells, including those found in the vagina. These channels regulate vital cellular processes such as cell volume regulation, fluid transport across epithelial layers, and muscle contraction [79]. Leak ion channels, a non-specific ion channel, play a crucial role in the electrical activity of smooth muscle, particularly in uterine smooth muscle during pregnancy [80].

The store-operated system is a fascinating mechanism in cells that maintains Ca^2+^ homeostasis. Upon the activation of intracellular Ca^2+^ reserves in the sarcoplasmic reticulum, a significant amount of Ca^2+^ will exit the cell. When intracellular Ca^2+^ stores are depleted, Ca^2+^ escapes from the cell, necessitating the refilling of these stores from the extracellular environment. Any depletion of these stores triggers Ca^2+^ influx into the cell. These channels responsible for this process are known as Ca^2+^ release-activated channels (CRAC). ICRAC, a subtype of the CRAC family activated by STIM proteins, exhibits unique properties by selecting Orai1 and Orai2 isoforms. It is non-voltage sensitive, inwardly rectifying, and highly selective for Ca^2+^. The conductance of ICRAC channels is extremely low, likely less than 1 pS. Interestingly, unlike other channels permeable to divalent ions, like Ca^2+^ and Mg^2+^, ICRAC’s selectivity is not influenced by the presence of these ions. Even in the absence of divalent cations, the channel’s conductance remains low. Furthermore, these channels can be selectively blocked by the trivalent ion Gd^3+^ and by the compound BTP2 from Synta [74]. 

According to several reports, the lower urinary tract, which includes VSM, consists of a set of ion channels, such as the voltage-gated Ca^2+^ channel (T and L type), the voltage-gated K^+^ channel (Kir, Kv1, Kdr), the voltage-gated Na^+^ channel, voltage-gated Cl^−^ channel Ca^2+^-activated K^+^ (KCa) channels (large conductance and small conductance), the K_ATP_ channel, the CRAC channel, the TRPM channel, and leak channels [81,82,83]. The K_ATP_, Kir, KA, and Kdr are ATP-gated, inward rectifying, A-type, and delayed rectifier-type voltage-gated K^+^ channels. The large and small conductance-based Ca^2+^-activated K^+^ channels are also known as BKCa and SKCa.

Interstitial cells of Cajal (ICCs) are unique cells that share a developmental origin with smooth muscle cells from mesenchymal cells [84]. While smooth muscle cells primarily focus on developing contractile structures, the ICC differentiates with fewer contractile elements but possesses abundant mitochondria, extensive endoplasmic reticulum, and specialized membrane channels. Structurally, the ICC features a spindle-shaped body with a slender cytoplasm, a prominent oval nucleus, and branching dendritic-like projections [85]. These dendritic processes further branch into secondary and tertiary extensions. Additionally, ICCs react positively to vimentin antibodies, distinguishing them from adjacent smooth muscle cells. ICCs are not limited to the gastrointestinal (GI) tract; they are also present in various organs and tissues, including the bladder, ureteropelvic junction, vas deferens, prostate, penis, mammary gland, uterus, pancreas, and blood vessels, such as the portal vein and vagina [84]. ICCs serve as the pacemaker cells of the GI tract by generating and propagating electrical slow waves. These slow waves, produced by ICCs, are regular depolarizations that trigger contractions in adjacent smooth muscle cells. This coordinated activity ensures rhythmic and synchronized contractions throughout the GI tract, facilitating the movement of food and waste [86]. It is also suggested that ICCs might play a similar role in VSM cells [87,88]. 

**Figure 3:**
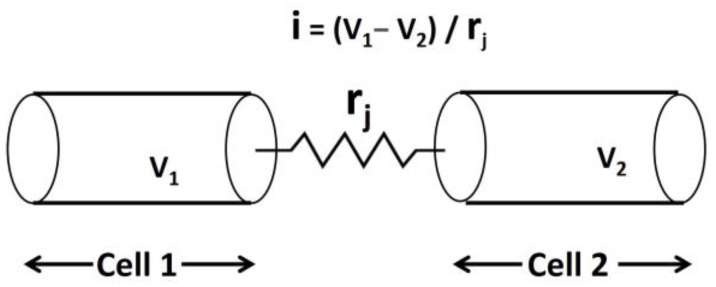
Illustration of the gap junction connection between two cells, Cell 1 and Cell 2. V_1_ and V_2_ are membrane potentials of Cell 1 and Cell 2, where the r_j_ value is the gap junction resistance between the two cells.

Smooth muscle cells are interconnected through specialized structures called gap junctions, facilitating direct cell-to-cell communication [89]. Connexin 26 (Cx26) is a protein encoded by the GJB2 gene and plays a pivotal role in forming gap junctions in excitable cells [90]. These gap junctions form electrical pathways of high conductance, allowing the flow of ions and small molecules between adjacent cells. This interconnected network of smooth muscle cells, enabled by gap junctions, creates a functional syncytium [91]. In a syncytium, electrical activity and responses are coordinated across multiple cells, allowing smooth muscle tissues to contract and relax in a coordinated manner. This property of syncytial behavior ensures efficient and synchronized muscle function in various physiological processes. Figure 3 illustrates the schematic representation of the gap junction connection between two cells, Cell 1 and Cell 2. V_1_ and V_2_ represent the membrane potentials of Cell 1 and Cell 2, respectively, while r_j_ denotes the gap junction resistance between the two cells.

The identification of Connexin 26 (Cx26) protein in the vagina supports the importance of gap junctions in facilitating the transmission of intercellular electrical activities in VSM cells [92]. 

Table 2 illustrates the role of various ion channels in smooth muscle AP/SW generation.

## 5. Calcium Dynamics in VSM Contraction

Calcium signaling pathways are pivotal in various cellular processes, serving as a universal signaling mechanism in eukaryotic cells [93]. These pathways regulate diverse functions such as muscle contraction, neurotransmitter release, gene expression, and cell proliferation and differentiation [94]. Intracellular Ca^2+^ levels are tightly controlled by a delicate balance between Ca^2+^ influx through various channels and Ca^2+^ efflux through pumps and exchangers. The dynamic changes in Ca^2+^ concentration are a molecular switch, triggering downstream signaling events that orchestrate cellular responses to internal and external stimuli [95]. The phenomenon, E-C coupling, involves linking alterations in membrane potential to intracellular Ca^2+^ concentration changes, ultimately leading to force generation. E-C coupling is explained in detail in the introduction section. Sarcoplasmic reticulum (SR) Ca^2+^ dynamics are pivotal in regulating smooth muscle contraction. The SR, a specialized organelle in smooth muscle cells, serves as the primary intracellular Ca^2+^ store and orchestrates Ca^2+^ signaling events crucial for contractility [96]. Dynamic changes in SR Ca^2+^ levels, governed by Ca^2+^ release and reuptake mechanisms, finely tune smooth muscle contraction and relaxation. Calcium release from the SR into the cytoplasm initiates muscle contraction by binding to contractile proteins, while subsequent Ca^2+^ reuptake into the SR via Ca^2+^-ATPase pumps facilitates muscle relaxation. This intricate interplay between SR Ca^2+^ release and reuptake ensures precise control over smooth muscle contractile activity, contributing to various physiological functions, including vascular tone regulation and organ motility [97]. The process of sarcoplasmic calcium-induced calcium release (CICR) is a critically important mechanism that regulates smooth muscle contraction, playing a vital role in numerous physiological processes. In smooth muscle, CICR is activated primarily when cytosolic Ca^2+^ levels surpass 1μM. This process entails L-type Ca^2+^ channels stimulating ryanodine receptors (RYRs), which results in the formation of Ca^2+^ sparks and the propagation of Ca^2+^ waves. These Ca^2+^ signals intensify the initial Ca^2+^ influx, thereby playing a vital role in regulating smooth muscle contraction and various cellular functions. Concurrently, inositol 1,4,5-trisphosphate (IP3)-induced calcium release (IICR) serves as another essential mechanism. IP3 acts as a second messenger, produced upon G-protein-coupled receptor activation, and subsequently binds to its receptor on the sarcoplasmic reticulum. This binding triggers Ca^2+^ ion release from intracellular stores. IP3-mediated Ca^2+^ release is integral to diverse cellular processes, such as muscle contraction, cell signaling, and neurotransmission. Moreover, adenine nucleotides can modulate the IP3-induced Ca^2+^ release in smooth muscle cells, highlighting the intricate regulation of this pathway [96,98]. Furthermore, the dysregulation of Ca^2+^ signaling pathways has been implicated in numerous diseases, including cancer, neurodegenerative disorders, and cardiovascular diseases [99]. For instance, aberrant Ca^2+^ signaling contributes to uncontrolled cell proliferation, apoptosis resistance, and metastasis in cancer [99]. Understanding the intricate mechanisms of Ca^2+^ signaling and its dysregulation in disease states holds promise for developing targeted therapies to restore cellular homeostasis and treat a wide range of pathologies [100].

## 6. The Model of Tension Generation in VSM Cells

**Figure 4:**
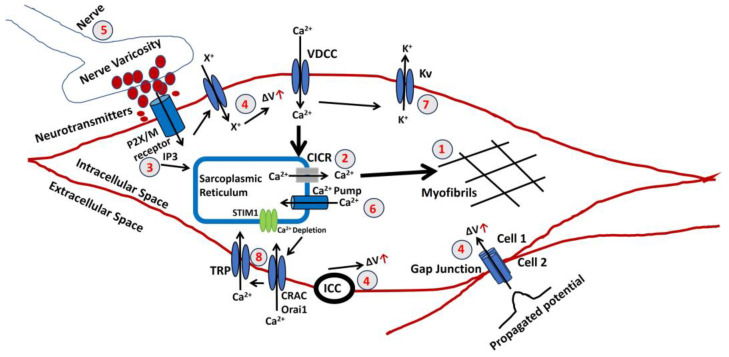
A schematic representation of the factors involved in contractile activation and relaxation of vaginal smooth muscle. The numbers refer to the steps explained in the text.

Figure 4 illustrates a schematic representation of the sequential processes entailed in the production of active tension within the vaginal smooth muscle. Both variables possess the potential for modulation and may play a role in the development of abnormal vaginal contractile function. Nevertheless, the pathophysiology of each step in the development of vaginal smooth muscle has not been thoroughly investigated. Instead, these findings are based on analyzing other smooth muscle models that have been experimentally validated. A red color number in the circle denotes the steps. The ΔV is known as a rise in membrane potential.

1. The final stage in generating tension involves an increase in the sarcoplasmic concentration of Ca^2+^. The myofibrils exhibit a comparable sensitivity to Ca^2+^, as shown in other muscles, necessitating a Ca^2+^ concentration of around one mmol/L for half-maximal activation. Ca^2+^ forms complexes with a soluble protein called calmodulin. This complex triggers a series of processes that activate a part of the myosin molecule by phosphorylation. As a result, actin and myosin can interact, requiring ATP. An elaborated explanation can be found in the introduction section.
2. Sarcoplasmic Ca^2+^ is derived from the SR, an intracellular reservoir. Ca^2+^ ions are transported from the storage site to the sarcoplasm by Ca^2+^ channels, which intracellular agents control. The formation of tension is influenced by various factors that affect the buildup or release of calcium in the SR. Any disruption to the cellular metabolic mechanisms that produce ATP would undermine their effectiveness. The release of Ca^2+^ from the SR can often be accomplished through one of two methods. An increase in the Ca^2+^ concentration near the SR triggers further release of Ca^2+^. The CICR mechanism is usually initiated by a Ca^2+^ flux across the surface membrane, although this is not always true.
3. There is a possibility of an elevation in the concentration of a diffusible second messenger, which connects the surface membrane with the release of intracellular Ca^2+^. The primary mechanism in a typical human vagina smooth muscle involves the binding of purinergic or acetylcholine (ACh) to the P2X or M3 muscarinic receptor, which triggers a series of membrane-bound processes, resulting in the synthesis of inositol trisphosphate (IP3). Alterations can significantly influence the release of intracellular Ca^2+^ in the sensitivity or gain of this mechanism.
4. Three reasons can cause a rise (denoted with a red arrow) in the membrane potential ΔV. The membrane potential can be propagated from Cell 2 to Cell 1 via the gap junction, as VSM behaves like a syncitium. Activating pacemaking cell ICCs can also trigger a rise in membrane potential. The extracellular ATP might bind to the purinergic receptor (P2X) and open a non-specific cation channel to permit the influx of any positive ion (X^+^), which can cause a rise in membrane potential. The resultant depolarization can open L-type Ca^2+^ channels, initiate Ca^2+^ influx, and trigger AP.
5. The parasympathetic nerves innervate the smooth muscle, and varicosites are the sites where the neurotransmitters are released. The number and distribution of excitatory nerves or the quantity of transmitter released modulate the membrane potential of VSM. The neurotransmitted might be purinergic or cholinergic cotransmitters.
6. The Ca^2+^ is filled in the SR lumen through a highly efficient ATP-dependent calcium pump, which transports calcium against a concentration gradient.
7. The decaying of the Ca^2+^ transient, which occurs after the generation of AP or SW, ceases VSM contraction. The activation of the Ca^2+^ channel and generation of AP/SW open the various K^+^ channels to repolarize the membrane, bringing the membrane potential to the RMP. After completing the contraction, the VSM cell returns to the relaxed state.
8. Store-operated calcium entry (SOCE) is a prevalent Ca^2+^ influx mechanism activated when intracellular Ca^2+^ levels in the SR drop, playing a role in regulating diverse physiological processes across various cell types. The TRP canonical (TRPC) channels, comprising TRPCs (1–7), are activated by stimuli that trigger PIP2 hydrolysis and were initially identified as key components of SOCE channels. TRPC channels exhibit a variety of tissue expressions, physiological roles, and channel characteristics. The search for the CRAC channel components led to the discovery of Orai1 and STIM1 as the primary elements of the CRAC channel. Substantial evidence now supports that STIM1 activates both Orai1 and TRPC1 through specific domains in its C-terminus. Interestingly, TRPC1’s function relies not only on STIM1 but also on Orai1. The essential functional interplay between TRPC1 and Orai1, crucial for TRPC1 activation, has been identified. This review will delve into the current understanding of TRPC channels in SOCE, the physiological processes governed by TRPC-mediated SOCE, and the intricate regulatory mechanisms of TRPCs, including their interactions with Orai1 and STIM1.

## 7. Experimental and Computational Techniques for Studying VSM Contraction

In vitro studies utilizing experimental techniques offer valuable insights into understanding vaginal smooth muscle contraction. These techniques involve the isolation of vaginal tissue strips, which are then subjected to controlled conditions to evaluate contractility. For instance, Cellai et al. conducted in vitro contractility studies on vaginal strips to assess the influence of testosterone on nitric oxide-induced relaxation [101]. By measuring the contractile responses under various experimental conditions, researchers can elucidate the signaling pathways and regulatory mechanisms involved in vaginal smooth muscle contraction. Such studies contribute to the development of pharmacological interventions targeting vaginal disorders associated with smooth muscle dysfunction. Furthermore, methodologies, such as inflation-extension testing, provide a comprehensive evaluation of vaginal tissue mechanics and contractility. Utilizing in vivo pressure measurements and biaxial testing, studies have explored the viscoelastic properties and contractile behavior of vaginal smooth muscle [6]. These experimental approaches enable the characterization of mechanical properties and responses to various stimuli, shedding light on the physiological and pathological mechanisms underlying vaginal function. Overall, in vitro studies employing experimental techniques play a crucial role in advancing our understanding of vaginal smooth muscle contraction and hold promise for future therapeutic strategies. In vivo, approaches utilizing experimental techniques offer valuable insights into understanding vaginal smooth muscle contraction within its physiological context. Studies have employed methodologies, such as in vivo pressure measurements and multiaxial loading, to evaluate the contribution of smooth muscle to vaginal viscoelastic response [6]. By conducting experiments directly within living organisms, researchers can assess the dynamic interactions between smooth muscle fibers and surrounding tissues, providing a comprehensive understanding of vaginal function and mechanics. Moreover, investigations utilizing in vivo models allow for studying smooth muscle contraction under physiological conditions, considering factors such as pregnancy and aging. These studies contribute to elucidating the role of smooth muscle in maintaining vaginal integrity and function throughout various life stages [6]. By combining in vivo experimental approaches with clinical observations, researchers can develop more effective therapeutic strategies for addressing vaginal disorders associated with smooth muscle dysfunction.

Computational modeling offers a powerful approach to studying vaginal smooth muscle contraction, allowing researchers to simulate and analyze complex physiological processes with precision. There are a lot of computational models established for different types of smooth muscle electrophysiology [15,102,103,104,105,106,107,108,109,110,111,112,113,114]. By integrating experimental data and physiological parameters, these models can elucidate the underlying mechanisms governing smooth muscle function in the vaginal wall. For instance, finite element analysis (FEA) has been employed to characterize the mechanical behavior of pelvic floor muscles during vaginal contractions, providing insights into the strains induced in the vaginal tissue [115]. These computational approaches enable researchers to explore various factors influencing smooth muscle contraction, such as hormonal changes, pregnancy, and aging, providing a comprehensive understanding of vaginal biomechanics.

Furthermore, computational models allow for the prediction of the effects of different interventions or pathologies on vaginal smooth muscle function. By simulating scenarios in silico, researchers can assess the efficacy of potential treatments for conditions like pelvic organ prolapse or urinary incontinence, guiding the development of targeted therapeutic strategies [115]. These models serve as valuable tools for both basic research and clinical applications, offering a cost-effective and non-invasive means of studying vaginal smooth muscle contraction and its implications for women’s health.

## 8. Clinical Implications and Future Directions

The pathophysiology of vaginal disorders holds significant clinical implications for women’s health. The therapeutic targeting of membrane potential and ion channels holds significant clinical implications across various medical fields. For instance, ion channels play crucial roles in cellular excitability, neurotransmission, and muscle contraction, making them attractive targets for pharmacological interventions [116]. Modulating ion channel activity can lead to the development of novel therapies for neurological disorders, cardiovascular diseases, and cancer [117]. Additionally, targeting mitochondrial ion channels shows promise in cancer therapy, offering a potential strategy to eliminate cancer cells selectively [118]. These advancements underscore the clinical relevance of understanding ion channel function and developing targeted therapeutics to address various pathological conditions. Looking ahead, future directions in therapeutic targeting of membrane potential and ion channels involve exploring innovative approaches to modulate ion channel activity with higher specificity and fewer side effects. Advancements in drug discovery technologies, such as high-throughput screening and computational modeling, enable the identification of novel ion channel modulators [119]. Moreover, personalized medicine approaches may facilitate the development of tailored ion channel-targeted therapies based on individual patient profiles, optimizing treatment efficacy and minimizing adverse effects. Future research directions in the pathophysiology of vaginal smooth muscle contraction aim to deepen our understanding of the mechanisms underlying pelvic floor disorders and to identify novel therapeutic targets. Investigating the molecular signaling pathways involved in regulating vaginal smooth muscle tone and contractility could provide insights into the pathogenesis of conditions such as pelvic organ prolapse (POP) and stress urinary incontinence (SUI) [120]. Additionally, exploring the role of hormonal influences, neurotransmitters, and extracellular matrix components in modulating vaginal smooth muscle function may offer new avenues for intervention and treatment strategies [10]. Furthermore, future research efforts could focus on developing innovative diagnostic techniques and therapeutic modalities targeting vaginal smooth muscle dysfunction. Advancements in imaging technologies, such as high-resolution ultrasound and magnetic resonance elastography, may enable non-invasive assessment of vaginal biomechanics and contractile function [14]. Moreover, developing precision medicine approaches tailored to individual patient characteristics and disease phenotypes holds promise for optimizing treatment outcomes and minimizing adverse effects [10]. Collaborative efforts between clinicians, researchers, and industry stakeholders are essential for translating these insights into clinically relevant applications, ultimately improving the management and outcomes of vaginal disorders.

## 9. Conclusions

The vagina plays a pivotal role in sexual intercourse, serving as a conduit for sperm to travel to the uterus and fallopian tubes for fertilization. Additionally, it functions as the birth canal during childbirth, facilitating the passage of the baby from the uterus to the outside world. Vaginal smooth muscle significantly contributes to regulating vaginal tone, thereby maintaining vaginal structure and function. Its contractile activity is essential for various physiological processes within the vagina, including supporting childbirth and sexual function. Fundamental research on vaginal smooth muscle contraction is crucial for understanding its role in vaginal viscoelasticity and structural integrity, which informs potential treatments for conditions like pelvic floor disorders. The study of electrophysiology in excitable cells enhances our comprehension of cellular function, uncovering ion channel biophysics, calcium dynamics, and neural control mechanisms as potential drug targets. However, smooth muscle electrophysiology research lags behind cardiac and neuronal electrophysiology due to challenges in isolating viable cells and applying electrophysiological techniques. Moreover, there lacks a single comprehensive review of vaginal smooth muscle electrophysiology to elucidate underlying ion channel biophysics and calcium dynamics. To address this gap, we present the first review of vaginal smooth muscle contraction electrophysiology, drawing from experimental, clinical, and computational studies to develop a model explaining the essential steps in vaginal smooth muscle contraction. These steps provide valuable directions for future research, enabling the exploration of new pharmacological targets for vaginal smooth muscle disorders.

## Figures and Tables

**Table 1:** Values of the RMP in various smooth muscle cells.

Smooth Muscle Type	RMP (mV)	AP/SW	Reference
Urinary bladder	−45 to −55	AP	[15]
Vas deferens	−60	AP	[53]
Ureter	−45	AP	[54]
Uterine	−50	AP	[55]
Urethra	−40	AP	[56]
Portal vein	−50	SW	[57]
Pulmonary artery	−55	SW	[58]
Aorta	−50	SW	[59]
Colon (GI tract)	−60	SW	[60]
Seminal vesicles	−50	SW	[61]

**Table 2:** Role of ion channels in AP/SW generations.

Ion Channel Type	Role in AP/SW
Ca^2+^ channels	Depolarization, RMP, AP firing
Na^+^ channels	Depolarization, AP firing
K^+^ channels	Repolarization, hyperpolarization, RMP
Cl^−^ channels	Depolarization, RMP
TRP channels	Depolarization, RMP, AP firing
Leak channels	RMP

## Data Availability

Not applicable.
